# C-Reactive Protein Monitoring Predicts Neutropenic Fever Following Autologous Hematopoietic Stem Cell Transplantation for Multiple Myeloma

**DOI:** 10.7759/cureus.2945

**Published:** 2018-07-08

**Authors:** Vidya Kollu, Sarah L Mott, Rafiullah Khan, Umar Farooq, Yogesh Jethava, Ince Dilek, Guido Tricot

**Affiliations:** 1 Internal Medicine, Medical College of Wisconsin, Milwaukee, USA; 2 Biostatistics/Holden Comprehensive Cancer Center, University of Iowa, Iowa City, USA; 3 Internal Medicine, University of Iowa, Iowa City, USA; 4 Internal Medicine, University of Iowa Hospitals and Clinics, Iowa City, USA

**Keywords:** c-reactive protein, adult, hematopoietic stem cell transplantation, multiple myeloma, febrile neutropenia

## Abstract

Background

Neutropenic fever (NF) is a known and common complication of autologous hematopoietic stem cell transplantation (ASCT). Early risk assessment may help direct treatment. We retrospectively analyzed the role of serial serum C-reactive protein (CRP) levels in predicting NF and assessed the clinical value of CRP within 14 days after transplantation.

Methods

One hundred twenty-one multiple myeloma (MM) patients received 170 first and/or second ASCT between January 2014 and March 2017. A Cox regression model was applied to assess the prognostic value of CRP as a time-dependent covariate at the onset of NF within 14 days post-transplant.

Results

Forty-seven of 170 patients developed NF. High CRP levels (4.0–43.2 mg/dL) were associated with a 5.45-fold increased risk of NF (P = 0.02). Patients had a nearly three-fold increased risk of NF after the second transplant (P < 0.01), but this was not associated with increased mortality. Those with NF had higher maximum values of CRP (P < 0.01) which tended to occur at or after the onset of NF.

Conclusion

CRP monitoring provides important information about the risk for NF immediately after first MM ASCT, and even more so after the second.

## Introduction

Multiple myeloma (MM) is characterized by the proliferation of clonal bone marrow plasma cells and a monoclonal immunoglobulin or light chain overproduction with evidence of end-organ damage [1]. MM represents 1.8% of all new cancer cases in the US annually [2] and is the second most common hematologic malignancy, affecting about four in 100,000 [3]. Hematopoietic stem cell transplantation (HSCT) refers to the administration of hematopoietic progenitor cells from any source (e.g., bone marrow, peripheral blood, umbilical cord blood) or donor (e.g., allogeneic, autologous) to promptly reconstitute bone marrow function after high-dose chemotherapy. Autologous HSCT (ASCT) uses hematopoietic progenitor cells derived from the patient. High-dose chemotherapy plus ASCT has been the standard treatment for newly diagnosed transplant-eligible MM adults up to 65 years of age [4]. In 2016, 8776 myeloma autologous transplants were reported to the Center for International Blood and Marrow Transplant Research. MM is currently the main indication for ASCT in Europe and the US [5, 6]. Tandem transplantation shows a higher overall survival (OS) and progression-free survival (PFS) when compared to patients who receive a single ASCT [7, 8].

After an ASCT, infection is the second most commonly reported cause of death, representing 15% of all fatalities, with disease relapse representing 69% of deaths [9]. Despite the many advances in supportive care, infections still continue to complicate the post-transplant course in many patients [10]; however, it is difficult to distinguish infection from other causes of fever, especially engraftment fever. The correct diagnosis of these major transplantation-related complications is essential for the early and proper administration of antibiotics or immunosuppressive treatment. The signs and symptoms of infection may be significantly muted in neutropenic patients or overlap with engraftment; thus, it is important to make use of objective evidence in the form of biomarkers to predict neutropenic fever (NF).

C-reactive protein (CRP) is an acute-phase protein produced by hepatocytes and is a reliable serum marker of infection, tissue destruction, necrosis and systemic inflammation [11-14]. While CRP testing is widely available and inexpensive, its use in day-to-day clinical decision-making is still limited and controversial.

Elevated CRP at diagnosis has a negative impact on myeloma prognosis [15]. Persistent elevation of CRP is an independent factor predicting fatal outcome in patients who remain febrile on the fifth day of antibiotic therapy during neutropenic febrile episodes post-HSCT [16]. CRP monitoring after blood and marrow transplant has identified patients at risk of severe transplant-related complications, mortality, and clinical outcomes [17, 18]. One recent study of the role of predictive biomarkers for cytokine release syndrome (CRS) after chimeric antigen receptor T cell therapy for acute lymphoblastic leukemia demonstrated that early CRP elevation was associated with grade 4–5 CRS (p = 0.02), but was not useful in predicting CRS severity [19].

While many studies highlight the importance of CRP in detecting major transplant-related complications following HSCT, accurate guidelines for CRP monitoring remain unsettled. To our knowledge, no studies have reported the relevance of CRP in patients receiving tandem transplants for MM on prophylactic broad-spectrum antibiotics. We report our experience with CRP monitoring and attempt to delineate the significance of CRP monitoring in patients undergoing tandem ASCT for MM on prophylactic antibiotics.

## Materials and methods

Study design

This retrospective study included 170 consecutive ASCTs for MM, received by 121 patients between January 2014 and March 2017 at the University of Iowa. The study was approved by the University of Iowa Institutional Review Board and performed according to best clinical practices. Inclusion criteria included first or second transplant and availability of at least one CRP level post-transplant. The evaluation period was day zero to day 14 post-transplant. All patients received dexamethasone, cisplatin, adriamycin and etoposide (DPACE) induction therapy before collection of CD34 stem cells [20]. The majority started conditioning chemotherapy with VDT-melphalan 200 mg/m^2^ on day four before transplant [21]. Seventeen patients received melphalan 140 mg/m^2^ due to decreased kidney function (stage IV), one patient received VD-busulfan, and three received VD-melphalan 200 mg/m^2^.

Prophylactic and conditioning regimens were systematically applied according to our protocol. Antimicrobial prophylaxis with ciprofloxacin, fluconazole, and acyclovir was started four days prior to transplant, at admission. On day five post-transplant, patients were started on prophylactic meropenem intravenously [22]. Cytomegalovirus (CMV) polymerase chain reaction and aspergillus galactomannan antigen were checked weekly. Patients received granulocyte-colony stimulating factor starting on day six. On day 12, darbepoetin alfa was given if hemoglobin was <10 mg/dL. During neutropenia, most patients remained hospitalized in an isolation unit with high-efficiency particulate air (HEPA) filtration.

Serum CRP levels were measured regularly during transplant, but only those obtained between days zero and 14 post-transplant were analyzed. Neutropenic fever was defined as having neutropenia and fever on the same day post-transplant. Neutropenia was defined as absolute neutrophil count (ANC) <500 µL or ANC level expected to fall below 500 over the next 48 hours. Prophylactic meropenem was used as a proxy for ANC expected to fall below 500 over the next 48 hours. Fever was defined as one temperature reading >101°F or at least two temperature readings >100.4°F. Engraftment was defined as ANC ≥ 500 for three consecutive days following HSCT. In the event of NF, blood cultures and chest X-ray were obtained and additional broad-spectrum antibiotics initiated. Additional microbiological and radiological tests were obtained as clinically indicated. Blood cultures (one of each lumen of a central catheter plus one peripheral culture) were performed only with the first fever. For subsequent fevers, a blood culture was drawn only once every 24 hours via catheter. The need for peripheral cultures was individualized by patient per hospital protocol. Of the 170 transplants, 46 did not have a single ANC value from days zero through 14 due to non-availability of ANC values when a patient’s total white blood cell count (WBC) was <1,000/µl attributable to lack of reliable reporting of a differential with such a low WBC count. Thus, NF was largely determined by additional intravenous antibiotic administration and elevated temperature. Of the remaining 124 transplants, the median number of ANC measurements was six. However, transplant patients had a median 13 CRP measurements. Temperature measurements were performed multiple times throughout the day, with median of 101 times per patient between days zero and 14.

Statistical analysis

Continuous variables were summarized using the median and range (minimum and maximum). Categorical variables were summarized by count and percent. Logistic regression models were applied to assess differences between NF groups. An exchangeable correlation structure was used to model the possible dependency among multiple transplants within a single patient. To assess the prognostic effect of CRP on onset of NF within 14 days post-transplant, a Cox regression model was utilized. Time was calculated from transplant to onset of NF. CRP was categorized by quartiles and analyzed as a time-dependent covariate. A robust sandwich estimator of the standard error was applied to account for a clustering effect of multiple transplants within a single patient. Estimated effects are reported as hazard ratios (HRs) along with 95% confidence intervals (95% CIs). A mixed-effects regression model was used to assess for differences in maximum CRP within 14 days post-transplant between those with and without NF. All tests were two-sided and assessed for significance at the 5% level using SAS v9.4 (SAS Institute, Cary, NC).

## Results

The study included 121 patients (94.2% were Caucasian, 5% were African American and 0.8% were Asian), 82 males (67.8%) and 39 females (32.2%). Median age was 61 years (range 38–76). Patient and transplant characteristics are summarized in Table 1.

**Table 1:** Patient characteristics and comparison of transplant characteristics by group. CMV: Cytomegalovirus; ASBMT: American Society for Blood and Marrow Transplantation; KPS: Karnofsky Performance Scale; ANC: Absolute Neutrophil Count.

	Neutropenic Fever		
Covariate	No N = 123	Yes N = 47	p-value
History of prior malignancy			
No	118 (95.9)	46 (97.9)	0.41
Yes	5 (4.1)	1 (2.1)	
CMV			
Negative	60 (48.8)	24 (51.1)	0.80
Positive	63 (51.2)	23 (48.9)	
Comorbidity index			
High	82 (66.7)	38 (80.9)	0.06
Intermediate-low	41 (33.3)	9 (19.1)	
ASBMT risk			
High	31 (32.3)	11 (26.2)	0.37
Low	65 (67.7)	31 (73.8)	
Preparative regimen			
VD-busulfan	1 (0.8)	0	—
VD-melphalan 200	2 (1.6)	1 (2.1)	
VDT-melphalan 140	12 (9.8)	5 (10.6)	
VDT-melphalan 200	108 (87.8)	41 (87.2)	
Transplant			
First	84 (68.3)	19 (40.4)	<.01
Second	39 (31.7)	28 (59.6)	
Disease status at transplant			
Complete response	31 (25.4)	20 (42.6)	0.05
Not complete response	91 (74.6)	27 (57.4)	
Performance status at transplant			
KPS	81 (66.4)	31 (67.4)	0.94
KPS-90	41 (33.6)	15 (32.6)	
Best response post-transplant			
Complete response	74 (73.3)	33 (82.5)	0.27
Not complete response	27 (26.7)	7 (17.5)	
New malignancy post-transplant			
No	121 (98.4)	45 (95.7)	0.32
Yes	2 (1.6)	2 (4.3)	
Age at transplant (years)			
Median	61	60	0.22
Range	38-76	38-76	
CD43 infused (million cells/kg)			
Median	8.1	8.7	0.10
Range	2.9-12.7	3.9-13.4	
ANC engraftment (days)			
Median	11	11	0.52
Range	7-28	10-19	

The median number of CD34 cells infused was 8.5 million/kg. The source of hematopoietic stem cells was peripheral blood in all cases. The median ANC engraftment day was day 11 (range 7–28). One patient with a high comorbidity index and low Karnofsky Performance Scale (KPS) died prior to engraftment during the second transplant due to infection and pulmonary toxicity. Of the 170 transplants, 47 developed NF (Table 1). NF occurred more often after second transplant (28 of 67, 42%) compared to first transplant (19 of 103, 18%). Though NF was more frequent after the second transplant, it was not associated with an increased mortality rate in the first 30 days. For patients who developed NF within 14 days post-transplant, engraftment occurred a median three days after the development of NF.

On multivariate analysis, high CRP levels (4.0–43.2 mg/dL) were associated with a 5.45-fold increased risk of NF (P = 0.02) compared to undetectable levels (<0.5 mg/dL, Table 2).

**Table 2:** Comparison of C-reactive protein (CRP) levels.

Variable	Comparison	Hazard Ratio	95% CI		p-value
CRP level	High (4.0-43.2) vs undetectable (<0.5)	5.45	1.32	22.6	0.02
	Low (0.5-4.0) vs undetectable (<0.5)	2.38	0.70	8.13	0.17
Transplant	Second vs first	2.98	1.73	5.13	<.01

**Figure 1:**
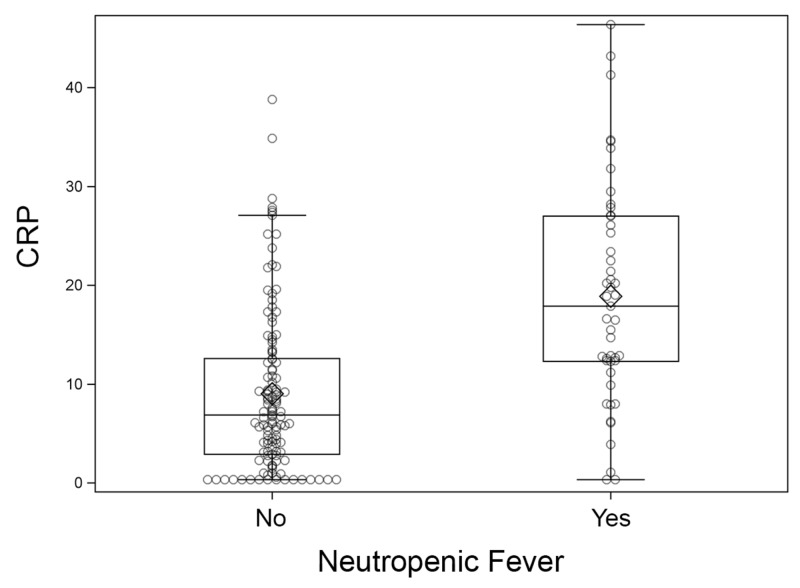
Maximum C-reactive protein (CRP) within 14 days of transplant.

Maximum CRP values were compared between those with and without NF. Patients developing NF post-transplant had higher maximum values of CRP compared to patients who did not develop NF (P < 0.01, Figure 1).

Among those who developed NF, the maximum value of CRP was more frequently observed at the time or shortly after the onset of NF (35 of 47, 74%).

## Discussion

Our study demonstrated that in the presence of prophylactic broad-spectrum antibiotics, high CRP levels (4.0–42.3 mg/dL) were associated with a 5.45-fold increased risk of NF (P = 0.02) compared to undetectable levels (<0.5 mg/dL) following ASCT for MM. Patients receiving the second of tandem autologous transplants were at nearly three-fold increased risk of NF compared to the first transplant. Additionally, while high CRP levels were predictive of NF, levels frequently continued to increase after the onset of NF. NF most often occurred before engraftment; engraftment occurred a median three days after the onset of NF.

Serial CRP monitoring has been found to be valuable for the early diagnosis and monitoring of infections in neutropenic febrile children [23] and has reliably differentiated between bacterial infections and other causes of fever [24]. In one study, CRP was shown to be useful in monitoring the response to therapy for NF episodes, but did not predict NF or differentiate between the causes of fever [25]. Most reports to date have included relatively few patients, with various underlying diseases and therapies, including conventional and myeloablative chemotherapy with autologous or allogeneic transplant. The real frequency of bacteremia in our patients could not be assessed accurately because all patients were on empiric meropenem from day five.

Investigators have evaluated the impact of CRP levels on clinical outcomes in allogeneic HSCT. Sato et al. found that higher baseline CRP level was an independent risk factor for non-relapse mortality (NRM; HR: 6.21, P < 0.01), grade III–IV acute graft versus host disease (HR: 3.91, P = 0.03) and poor overall survival (HR: 3.27, P = 0.0018) [26]. Pavlu et al. reported that higher CRP levels independently predicted inferior survival and increased NRM in allogeneic HSCT for chronic myeloid leukemia [27]. Previous studies have examined inflammatory factors related to the development of high symptom burden during ASCT for MM. Wang et al. reported that serum CRP was significantly related to the most severe symptoms during the first 30 days after ASCT (P < 0.05) [28]. Fassas et al. demonstrated that a cut-off point of 100 mg/L (10 mg/dL) CRP and 15 mg/L/day (CRP velocity) identified patients likely to suffer severe complications with 86% and 75% sensitivity, respectively [29]. Significantly higher mean CRP levels and CRP velocity of increase have also been observed among patients with severe complications [23]. Sato et al. reported that CRP levels immediately prior to first consolidation chemotherapy, but not before induction chemotherapy, had a significant predictive value for febrile neutropenia at a cut-off value of 0.19 mg/dL and documented infection at a cut-off value of 0.26 mg/dL [30].

Our study has certain limitations related to its retrospective nature. While high CRP levels were significantly associated with NF, we could not assess whether elevation in CRP could reliably distinguish between engraftment fever versus infection.

## Conclusions

Early indication of NF helps identify those patients more likely to benefit from a combination of antibiotics while reducing unnecessary toxicity in those with low risk for NF. CRP monitoring provides important information about predicting NF during ASCT for MM, more so in tandem transplant. High CRP levels (4.0–43.2 mg/dL) were associated with a 5.45-fold increased risk of NF (P = 0.02). Patients had a nearly three-fold increased risk of NF after the second transplant (P < 0.01), but this was not associated with increased mortality. Future prospective studies will help to elucidate the definitive role of CRP in identifying transplant patients at risk for NF and distinguishing engraftment fever from infections. Such results could be extrapolated to other transplant protocols for hematological malignancies.
